# Lessons of the Elwha River: Managing Health Hazards During Dam Removal

**DOI:** 10.1289/ehp.120-a430

**Published:** 2012-11-01

**Authors:** Wendee Nicole

**Affiliations:** Freelance science writer and photographer **Wendee Nicole** (formerly Holtcamp) has written for *Nature*, *Scientific American*, *National Wildlife*, and other magazines.


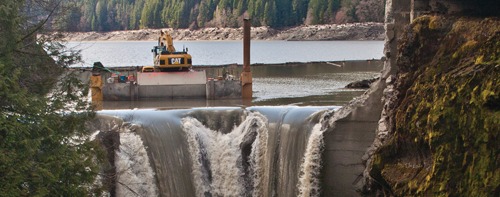
On the northwest edge of the continental United States, in some of the quietest and most rain-drenched lands in all of North America,^^1^^ runs the glacier-blue Elwha River. It arises from the Elwha Snowfinger, a perennial snowfield in Washington’s Olympic National Park, and flows 45 miles northward through basalt canyons and old-growth forest before spilling into the Strait of Juan de Fuca. The river traverses the reservation of the Lower Elwha Klallam Tribe, a people who have relied on the river’s salmon for physical, spiritual, and cultural sustenance for millennia.^^2^^

The river’s two hydroelectric dams have become stars of a four-decade-long saga, culminating in the complete removal of the lower Elwha Dam this past summer, with the taller Glines Canyon Dam breached but not yet gone; it should be fully removed by summer 2013. Together they make up the biggest dam-removal project and the second biggest restoration project ever undertaken by the National Park Service (NPS), after the Everglades.^^3^^ With the lower Elwha Dam gone, the Elwha River ecosystem—as well as the local tribal community—has begun a dramatic transformation.

**Figure:**
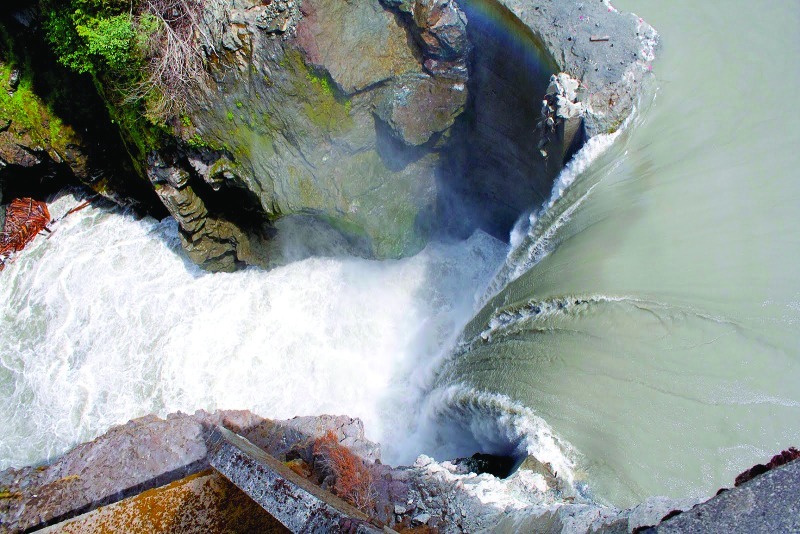
Deconstruction of the Glines Canyon Dam should finish in summer 2013. Tearing down dams restores ecosystems in the long term, but in the short term it releases mass quantities of pent-up sediment and, in some cases, contaminants. As Chinook salmon and other fish begin returning to Washington State’s Elwha River, the biggest dam removal in history in terms of dam size is being touted as a model for future dam breaches. © 2012 Wendee Nicole

This summer Chinook and other salmon species spawned in tributaries that had been blocked for a century. Meanwhile, more than 24 million yd^3^ of clay, silt, sand, gravel, and cobble that had built up behind the dams began to flow.^^4^^ This sediment, especially the gravel, is necessary for the restoration of fish spawning habitat, and downstream beaches and stream beds long starved of gravel, sand, and silt will ultimately be bolstered by its return. But in the short term, excess turbidity remains the biggest concern for the watershed’s human and animal residents during the next 3–10 years.^^5^^^^,^^^^6^^ Dramatic increases in turbidity are expected to kill fish and diminish spawning success^^7^^ as well as affect water for drinking, hatcheries, and a paper mill. Negotiating how to mitigate these concerns took decades—and a lot of money.

Tearing down dams releases mass quantities of pent-up sediment and, in some cases, contaminants in the short term, but it restores ecosystems in the long term. As the fish begin returning to the Elwha River, the biggest dam removal in history is being touted as a model for future dam breaches.

## Back to the Future

The dams originated in the mind of entrepreneur Thomas Aldwell, who envisioned power plants to fuel the local economy and run a paper mill. “When these dams were built, [they provided] the only power on the Olympic peninsula,” says NPS public information officer Barb Maynes. The 105-foot Elwha Dam was completed in 1914 at river mile 4.9, and the 210-foot Glines Canyon Dam followed in 1927 at mile 13.6.^^8^^ Dam ownership changed hands over the years, but the relicensing processes for the two dams in 1968 and 1973, respectively, spurred what would become decades of debate.

In the 1970s, Robert Elofson, the Lower Elwha Klallam Tribe’s river restoration director, hiked to the Elwha headwaters. “I thought, ‘This river is gorgeous. All it needs is salmon,’ ” he says. At the time, he says, he imagined getting salmon past the dams, but never dreamed he would see those dams removed in his lifetime.

When Aldwell built the Elwha Dam, Native Americans were not considered U.S. citizens. “In the old days, we were physically defending our land,” says Elofson. “We still do, but in another way.” Now full citizens with enforceable tribal treaty rights to half the harvestable catch in their traditional fishing grounds, the tribe played a leading role in the decades-long battle to breach both dams. Environmental groups joined the fight to remove the dams in order to restore the river’s fisheries.

**Figure:**
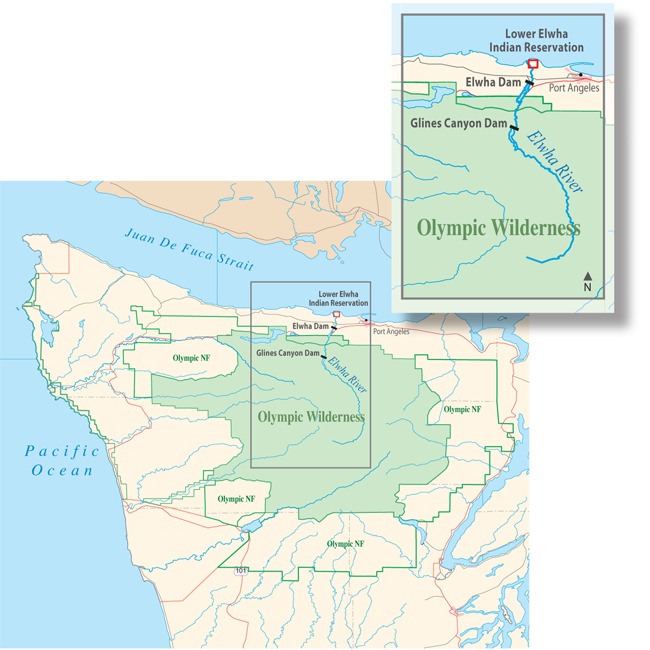
The Elwha River empties out of the Olympic Peninsula, in the northwest corner of Washington State. The Elwha River watershed includes more than 100 miles of tributaries and a 270-square-mile drainage area. © Map Resources/Joseph Tart/EHP

The dam owners initially balked, and many local townspeople feared a federal land grab, job losses, fouled water, and the removal of dams that were an integral part of local culture. The struggle eventually led to the Elwha River Ecosystem and Fisheries Restoration Act of 1992,^^9^^ a negotiated settlement. The act did not mandate removal of the dams but rather the “full restoration of the fisheries and ecosystem.” However, a 1995 environmental impact statement (EIS) determined that removing both dams was the only means to that end.^^10^^

“Once the secretary [of the Department of the Interior (DOI)] made his decision, that’s when the public came out in opposition,” says Brian Winter, Elwha project team leader for the NPS, which now owns the dams. “Dam removal was not as much in front of people’s minds as it is today.” On the other hand, he says, people were anxious for work, and the project offered local employment: “I’d go to businesses, and [people] would say, ‘I’m really against the project, but if you’re going to do it, do it soon because we need jobs.’”

“Last year when we shut down the power plants, it was a big deal,” says Richard Bauman, manager of the Elwha project for the U.S. Bureau of Reclamation (USBR), which was tasked with dam removal and preparing the site for demolition. “It was kind of emotional, stepping from one age into a new age.”

For the Elwha people, dam removal represents a return to a bygone age. The river once boasted famed runs of Pacific Northwest salmon species—Chinook, pink, chum, coho, and sockeye—as well as steelhead and cutthroat trout. People caught 100-pound Chinook here, and elders tell of walking across the backs of spawning salmon.^^6^^ The dams were built without fish passage devices even though such devices were mandated by an 1890 state law.^^11^^

**Fish Consumption**

Levels of PCBs measured in Elwha River fish in 1999 were relatively low—about 9–17 ppb total PCBs—but they exceeded the human health criterion of 5.3 ppb that Washington State uses for listing under 303(d), says Art Johnson, an environmental scientist with the Washington Department of Ecology (WADOE). Background levels of total PCBs in Washington freshwater fish fillets average around 5 ppb, while fish fillets from urban/industrial lakes and rivers in the state average around 60 ppb.^28^

“A lot of time when people find out a river is on the 303(d) list, they panic,” says WADOE environmental specialist Brandee Era-Miller. But risk, she says, is a function of consumption, and Native American populations whose diet relies heavily on fish are at greater risk of PCB-related health effects than the average person. The Washington Department of Health declares fish consumption advisories for PCBs at concentrations of 23 ppb and has set reference doses for subsistence fishers of 2.5 ppb for cancer and 9.83 ppb for noncancer end points;^28^ this represents the daily dose a subsistence fisher is estimated to be able to consume without appreciable risk of negative health effects.

Washington is in the process of revising its fish consumption rate, a value that represents the average amount of fish consumed statewide and that’s used to estimate exposure to toxics found in fish. The rate gets plugged in to risk assessment formulas that determine acceptable levels of human exposure to various compounds, which in turn determines what levels of pollutants are allowed in industry effluent.

“We feel the state fish consumption number is grossly inadequate,” says Matt Beirne, environmental coordinator for natural resources for the Lower Elwha Klallam Tribe. “It’s not protective of even the average fish consumer, let alone Native Americans.” The present state consumption rate is 6.5 g/day, but the Lower Elwha Klallam Tribe’s fish consumption falls in line with that of the Suquamish Tribe, the highest in the state at nearly 500 g/day, he adds.^29^

Washington is now revising its human health–based criteria water quality standards, which “are directly tied to pollution limits,” says Sandy Howard, WADOE communications manager. As the state revises its surface water quality standards for human health, even lower PCB levels will be required for a waterway to be declared clean.^30^

In 2011 Oregon became a national leader by raising its fish consumption rate from 17.5 g/day to a more realistic 175 g/day, resulting in the most protective water quality regulations in the United States.^31^ Washington State was set to establish new rates in August 2012, but WADOE director Ted Sturdevant opted to open the process up to more public comment.^30^^,^^32^ A draft technical document preliminarily recommends a fish consumption rate for Washington of 157–267 g/day.^33^

The health benefits of eating fish should be considered along with the risks.^34^ WADOE environmental scientist Keith Seiders says, “Folks get scared away from fish and turn to foods that may be more harmful,” including highly processed foods. “The risk of getting cancer [or other illnesses] from contaminated fish is very small,” Seiders says. “For some people, it’s more important to have their traditional culture and an increased risk of some effect from [toxics] in fish than for them to lose their cultural identity and way of fishing.”

**Figure:**
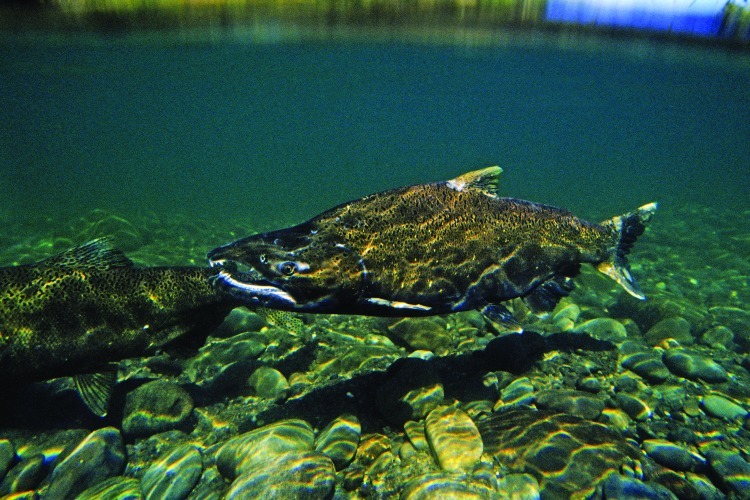
A Chinook salmon swims in the Elwha River © Natalie Fobes/Science Faction/Corbis

## Protecting the Water

The Elwha Act stipulated that municipal and industrial water must be protected in the event of dam removal. That resulted in a new water treatment plant for nearby Port Angeles, new wells for local water associations, and a new hatchery and wastewater collection system for the tribe. Once it was determined that the dams must go, plans were made through the EIS process to safeguard against increased flood risk with bolstered levees and to clean up lead, asbestos, polychlorinated biphenyls (PCBs), and petroleum hydrocarbons at the dam sites. Biologists would conduct pre- and postremoval studies of sediment transport to understand how it would affect both people and the environment, and the tribe would receive $4 million from the DOI for land acquisition and economic development for losses suffered from the dams.

Yet federal funds are never guaranteed, especially in lean economic times. “We weren’t confident [removal] was going to happen until the first contract was awarded,” says Elofson. Fifteen years after the passage of the act, construction finally began on the Port Angeles water treatment facility.

During the dam-removal planning process, the Washington State Department of Health determined that the city’s water source was not “groundwater” but “groundwater under the direct influence of surface water.” This meant the water was vulnerable to microbial contamination and needed more stringent protection to meet the EPA Surface Water Treatment Rule under the Safe Drinking Water Act.

The city would have had to build a facility with or without dam removal, but “the timing of it meant that when the Park Service constructed the city’s municipal water treatment plant, it had to address that [additional concern],” explains Maynes. She says the city’s new state-of-the-art facility plus an industrial water treatment plant cost the project more than $100 million.

Less than 10% of the project’s final $325 million budget^^12^^ was spent on dam removal and sediment monitoring, according to Tim Randle, USBR engineering manager. “We have new water treatment plants. We dug new wells for two municipal water associations, and raised the levees,” Randle says. “Did we have to spend all that money? We will get to an answer of that at the end of the removal.”

Once the water treatment facilities were operational, dam removal began. Randle and others had planned long and hard how best to remove the dams: quickly or slowly? “People joked, ‘we could get the Navy out of Bremerton^^13^^ to blow up the dams instantly.’ But the flood would come down, and the sediment would bury any survivor,” says Randle. “If you remove it really slow, one foot per year, you’d hardly notice the difference, but who wants to wait two hundred years to remove a two-hundred-foot dam? We came up with a viable option to remove it in controlled increments, and so far that plan is working really well.”

By May 2012 the Elwha Dam and its reservoir, Lake Aldwell, were no more. As of July, the Elwha River carried half a million tons of sediment, 50,000 standard dump-truck loads, according to Chris Magirl, a research hydrologist with the U.S. Geological Survey (USGS). “Only about five percent of the anticipated sediment load has been mobilized,” Magirl says. “So, in other words, the sediment party has just started.”

“This is the largest-ever controlled sediment release in a dam-removal project anywhere in the world,” says Randle. Dozens of scientists will continue to study water quality and the ecosystem, wading in the river’s tributaries to measure turbidity, scuba diving to catalog riverine life, tracking salmon, and monitoring how turbidity affects the water treatment facility, which supplies not just the city’s drinking water but also the paper mill and the state and tribal hatcheries.

Already, the turbidity has had unanticipated effects on the tribe’s hatchery. “[The hatchery] is doing its job, but the Elwha fish are even more sensitive than we thought they would be to sediment in the river,” Elofsen says. He says the salmon’s growth was impaired when the water hit 30 nephelometric turbidity units (NTU; a unit of turbidity), peaking in May 2012, although the hatchery water turbidity has since declined.

The Port Angeles facility was designed to process up to 40,000 mg/L total suspended solids^^14^^ (roughly 20,000–30,000 NTU, says Randle), but it remains to be seen how it will handle larger “sediment slugs” that will arise as further sections of the remaining dam are removed or with large rain events.

The only stakeholder still awaiting funds is the tribe; although their new hatchery and wastewater treatment facility are complete, they have not seen the promised $4 million. Former U.S. senator Bill Bradley (D–NJ), one of the Elwha Act’s sponsors, wrote a letter to DOI secretary Ken Salazar in March 2012, stating, “[W]e did not consider removal of the dams and restoration of the fisheries to be, by themselves, commensurate with the fulfillment of the nation’s obligation to the tribe.”^^15^^ But it is unclear whether or when this payment will be made. Elofson says the tribe is also still negotiating with the NPS on money for hatchery operations and maintenance.

## Environmental Contaminants

Cleaning up environmental contaminants at the two dam sites has received less attention than the sediment control aspect of the project. The structures associated with the dams were constructed when lead-based paint and asbestos were commonly used and when transformer oil contained PCBs. Testing prior to DOI acquisition of the dams had indicated that the transformer oil from both dams was PCB-free,^^16^^ so inspectors with the NPS were surprised to find 3,905 gallons of PCB-tainted oil in a couple of the transformers. The contaminants needed to be remediated to a level suitable for recreational use under state and federal environmental laws.

**Figure:**
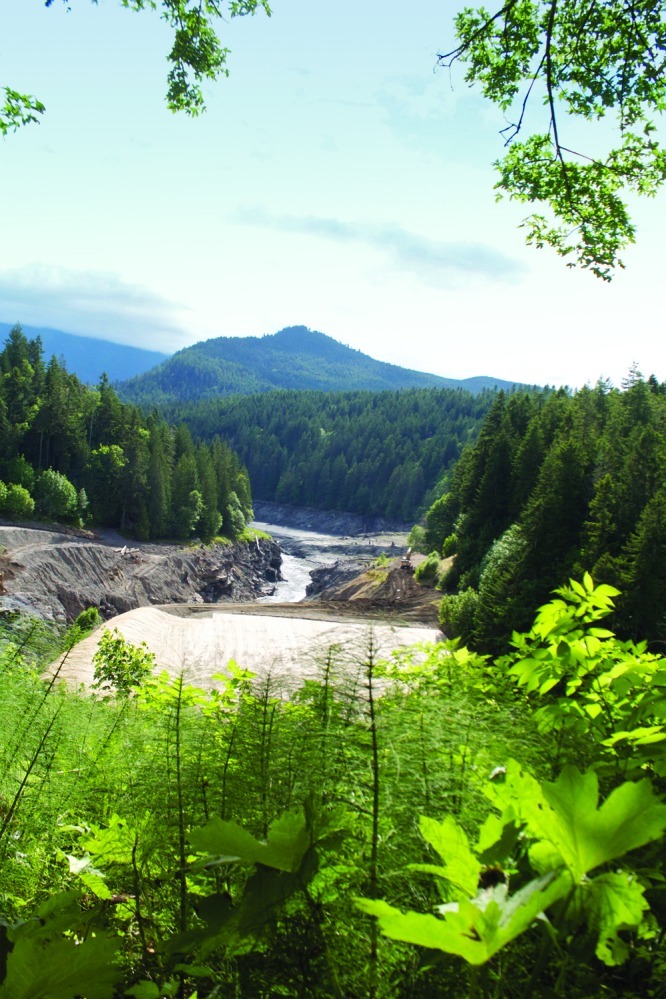
Workers restore habitat in what was once Lake Aldwell after removal of the Elwha Dam was completed in summer 2012. © 2012 Wendee Nicole

Contractors removed 1,000 yd^3^ of lead- and PCB-contaminated soil, which was disposed of at a facility in Oregon.^^15^^ Some lead-contaminated soil remains on site with concentrations up to 250 ppm; by comparison, the U.S. Environmental Protection Agency (EPA) has identified 400 ppm as its hazard standard for bare soil in play areas.^^17^^ Bauman says the USBR capped the remaining contamination with 15 feet of clean soil. Workers removed all asbestos, hauled off the PCB-contaminated oil, and recycled as much of the concrete and metal debris as possible.

The seemingly pristine river had long been on the EPA’s 303(d) list of waterways in violation of the Clean Water Act for PCBs—a site located between the two reservoirs tested positive for PCBs in the 1980s. Once a site is listed, it stays on that list until followup testing proves the condition no longer exists. So in 1999 researchers with the Washington Department of Ecology (WADOE) returned to test fish downstream of the lower dam.^^18^^ All composite samples of captured fish exceeded criteria for safe consumption for two PCBs under the EPA’s National Toxics Rule. As a result, under the Clean Water Act, the lower Elwha Dam was designated a category 5 water body, mandating a cleanup plan.

Despite the Elwha Act’s focus on fisheries and ecosystem restoration, the multimillion-dollar, multidecade project contained no provisions for addressing the 303(d) listing and ascertaining whether the dam-removal process might disturb PCBs in sediment. Instead, the 1996 draft EIS states simply that NPS policies require park managers to “take action to address existing hazardous waste problems such as . . . contamination of water, soil, and air.”^^5^^

Winter is satisfied with testing completed during the dam-removal process. “We [took] samples of the deltas because that’s the stuff that’s eroding, and we found no contaminants,” he says.^^19^^ Sediment from Lake Mills (the reservoir for the Glines Canyon Dam) was tested in 1994 during a “drawdown” experiment (in which the lake was drained to simulate dam removal),^^19^^ and three sediment samples were taken from Lake Aldwell in 1993,^^16^^ with no PCBs detected.

Although PCBs were found in the onsite soil and in operating components of both dams, fish contamination in the lower river could also be the result of atmospheric deposition or contaminated hatchery feed, a problem in the 1990s.^^18^^Alternatively, an unconfirmed rumor holds that a transformer fell in the river years ago. “A landowner who wanted to develop an RV park on private land within Olympic National Park installed a transformer as a part of his project,” says Winter. “Decades ago, a flood resulted in erosion of his land and loss of the transformer into the river, so the story goes.” The landowner’s site was located above Lake Aldwell.

Winter does not agree that the 303(d) listing is relevant to the Elwha project. “The responsibility for sampling, monitoring, and reporting to the public any possible health threat from PCBs rests with the water quality regulatory agencies, in this case WADOE,” he says. “Had there been any issue of PCBs and the restoration project by WADOE or the EPA, such a sampling requirement would have been included in the water quality certificate the NPS obtained.”^^20^^

The Fish Restoration Plan developed by the National Oceanic and Atmospheric Administration pursuant to the Elwha Act does mention the 303(d) listing and recommends regular water quality monitoring.^^21^^ “If people find these concentrations, we should be measuring them over time,” says administration fisheries biologist George Pess, who studies sediment impacts on Elwha fish. “It should be part of the landscape monitoring program.”

Thus far, however, it is not. And many scientists working on the project were unaware of the listing. “There are so many people doing so many different things,” Pess says, “I think it’s a case of one hand doesn’t know what another is doing.”

## Return of the Kings

Just as dam removal was set in motion, controversy struck again. In February 2012 four conservation groups sued the Lower Elwha Klallam Tribe and several federal agencies to halt the release of non-native steelhead trout from the tribe’s hatchery and Chinook salmon from the state hatchery.^^22^^ The tribe planned to use hatchery-reared fish to expedite recovery, especially during the period of heavy sediment load. The plaintiffs argued that hatchery-reared fish harm the reproductive fitness and genetic diversity of wild populations, particularly Puget Sound Chinook and native steelhead—both federally threatened species—and that fish will recolonize naturally. Shortly afterward, the tribe entered an interim agreement not to use the non-native hatchery-raised steelhead; the suit is ongoing.^^23^^

“Things are happening on a daily basis,” says Pess. “Numerous fish have spawned above the dam now.” This year, Chinook, pink, and coho salmon plus steelhead have spawned in river reaches previously blocked. The river ecosystem is undergoing a dramatic transformation and is a living laboratory for scientists. The people of the Lower Elwha Klallam Tribe have voluntary relinquished their rights to fish for five years, but in a few years they expect to see increased salmon runs in their historic fishing ground, as will recreational fishers. Most of the environmental contaminants from the dams will have been remediated and removed by then as well. Yet with the biggest sediment slugs still to come, the salient question for the restoration project is to quantify the impacts of these heavy sediment loads on spawning and rearing salmon.

If the Elwha tells a story, perhaps it is that ultimately it costs less to remove a dam and restore a river than it does to maintain the old dam.^^24^^ And despite intense controversy, people can eventually find accord, and diverse stakeholders can forge plans that satisfy most concerns.^^5^^^^,^^^^25^^ With less than 1% of America’s river miles protected in their natural state, and more than 80,000 large dams^^26^^ in the United States alone,^^27^^ more stories like this will surely unfold as infrastructure ages.

“I do think most people now see this as something very positive. It’s bringing a lot of attention to the area, which is good in terms of tourism,” says Maynes. “The dams are gone, fish are being seen in the river, and new beaches are establishing down at the mouth of the river. We’re already seeing benefits.”
